# Three-dimensional printed calcium phosphate scaffolds emulate bone microstructure to promote bone regrowth and repair

**DOI:** 10.1007/s10856-024-06817-8

**Published:** 2024-09-03

**Authors:** Kyohei Takase, Takahiro Niikura, Tomoaki Fukui, Yohei Kumabe, Kenichi Sawauchi, Ryo Yoshikawa, Yuya Yamamoto, Ryota Nishida, Tomoyuki Matsumoto, Ryosuke Kuroda, Keisuke Oe

**Affiliations:** 1 Department of Orthopaedic Surgery, Kobe University Graduate School of Medicine, Kobe, Japan; 2 Department of Orthopaedic Surgery, Hyogo Prefectural Nishinomiya Hospital, Nishinomiya, Japan; 3 Visiting Medical Scientist, Kobe University Graduate School of Medicine, Kobe, Japan

## Abstract

The interconnected structures in a 3D scaffold allows the movement of cells and nutrients. Therefore, this study aimed to investigate the in-vivo bioactivity of 3D-printed β-tricalcium phosphate (β-TCP) and hydroxyapatite (HAP) scaffolds that replicate biological bone. This study included 24-week-old male New Zealand white rabbits. A cylindrical bone defect with a diameter of 4.5 mm and a depth of 8 mm was created in the lateral aspect of the distal femur. A 3D-printed scaffold was implanted in the right femur (experimental side), whereas the left femur was kept free of implantation (control side). Micro-CT analysis and histological observations of the bone defect site were conducted at 4, 8, and 12 weeks postoperatively to track the bone repair progress. No evidence of new bone tissue formation was found in the medullary cavity of the bone defect on the control side. In contrast, on the experimental side, the 3D scaffold demonstrated sufficient bioactivity, leading to the growth of new bone tissue. Over time, new bone tissue gradually extended from the periphery toward the center, a phenomenon evident in both micro-CT images and biopsy staining. In the current study, we observed that the cells involved in bone metabolism adhered, spread, and proliferated on our newly designed 3D-printed scaffold with a bone microstructure. Therefore, it is suggested that this scaffold has sufficient bioactivity to induce new bone formation and could be expected to be a more useful artificial bone than the existing version.

**Graphical Abstract:**

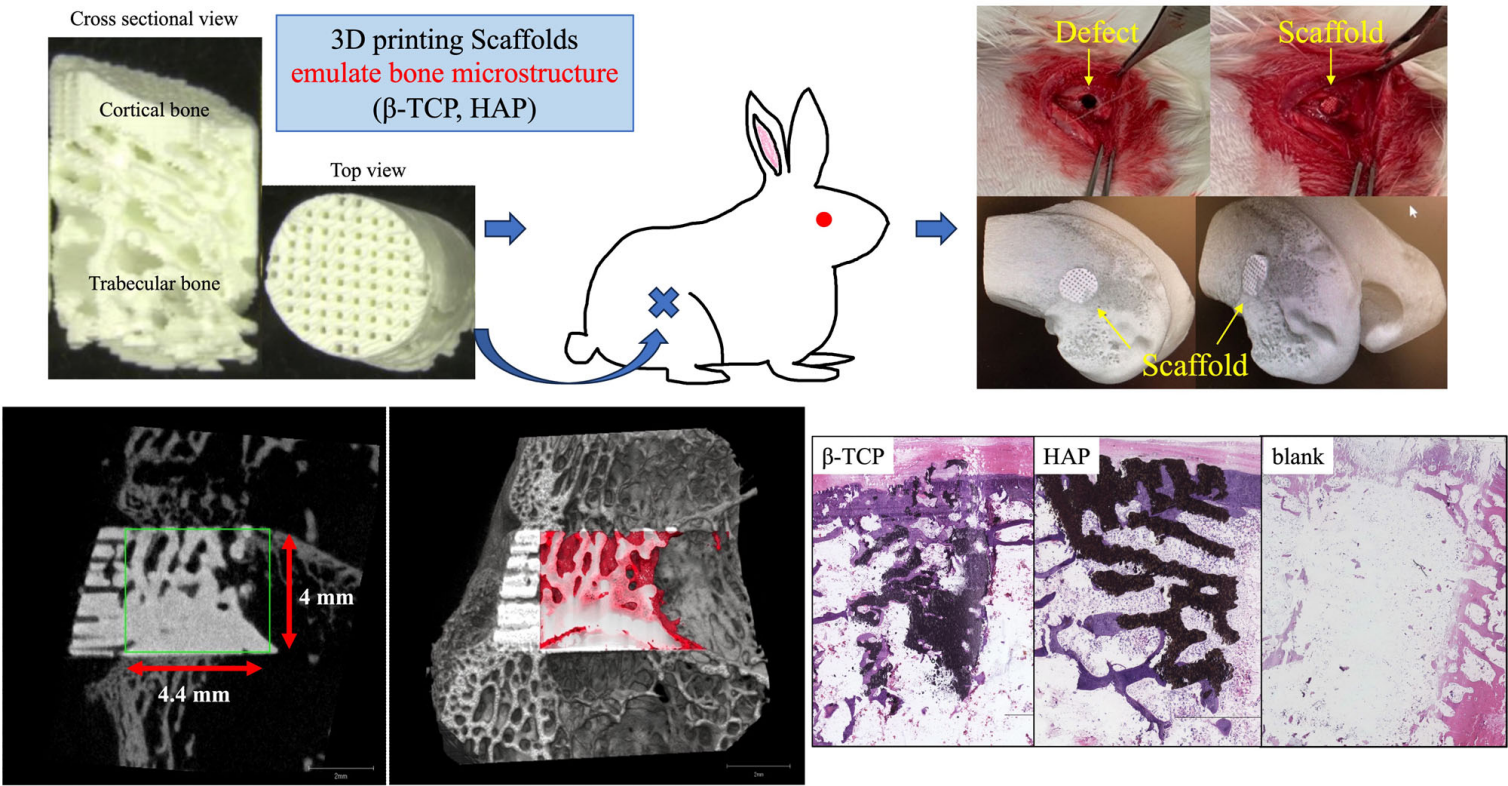

## Introduction

The treatment of large bone defects presents a clinical challenge for orthopedic surgeons. Although autologous bone grafts and allografts are widely used as the gold standard for the treatment of bone defects, they have several drawbacks, including donor-site morbidity, a limited quantity of grafts, and disease transmission [1, 2]. Artificial bones have been developed to overcome these problems using autogenous and homologous bones.

Calcium phosphate materials such as hydroxyapatite (HAP) and β-tricalcium phosphate (β-TCP) are often used as artificial bone in daily clinical practice. These materials exhibit excellent biocompatibility and osteoconductivity [3, 4]. HAP artificial bone resorbs similarly to biological bone; however, the resorption rate is very slow, so it remains semi-permanent in the body and virtually non-resorbable [5]. A bioabsorbable artificial bone made of β-TCP, which has higher solubility and is easily absorbed compared to hydroxyapatite, has been developed and widely used clinically, as it is dissolved and resorbed in the acidic environment created by osteoclasts like biological bone [6–8]. Currently, the most common approach for creating scaffolds with osteoinductive properties is to incorporate growth factors such as bone morphogenetic proteins (BMPs) into the material [9, 10]. Using natural extracellular matrix (ECM) proteins such as collagen, which can support the growth of new bone tissue, is another approach [11, 12]. ECM proteins can be combined with growth factors or other bioactive molecules to enhance their osteoinductive properties. Researchers are also exploring the use of stem cells to create those scaffolds [9].

Many recent calcium phosphate artificial bones have extremely high porosity, and there is a tendency to emphasize bone ingrowth by increasing the scaffold surface area at the expense of the initial strength. These materials are primarily in the shape of granules or beads, making it difficult to precisely match the shape of the bone defects. Three-dimensional (3D) printing, also known as additive manufacturing, has been studied for several decades. In recent years, this technique has advanced in the field of tissue engineering and has been used to repair damaged tissues [10, 13], enabling the production of scaffolds with arbitrary shapes [14–16] and 3D structures with complex internal morphologies, such as trabecular bone. In other words, a customized implant that fits the specific shape of a patient’s bone can be manufactured using a 3D printer according to the patient’s requirements. Other features include adjusting porosity, introducing interconnected pore structures, and facilitating shape and size control [14, 15].

A 3D scaffold should have interconnected structures that allows the movement of cells and nutrients. Considering the advantage of a 3D structure for bone regeneration, we hypothesized that bone regeneration would be improved by the implantation of a 3D scaffold with the trabecular structure of biological bone, facilitating cell and fluid penetration. This study aimed to investigate the in-vivo bioactivity of 3D-printed β-TCP and HAP scaffolds that replicate biological bone.

## Materials and methods

### Scaffold design and fabrication

**Fig. 1:**
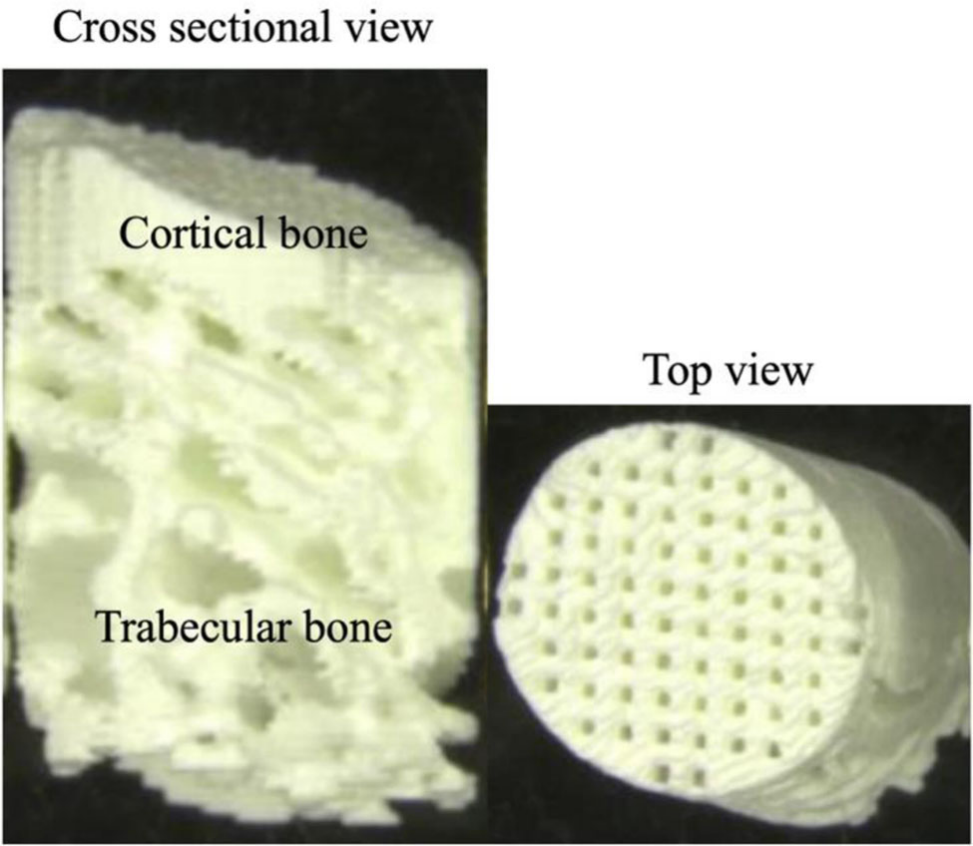
Structure of the 3D-printed scaffold. The trabecular bone area was emulated as biological bone, and the cortical bone area was a dense body with bony pores

In this study, the pure β-TCP and HAP were provided by Tomita Pharmaceutical Co. Ltd. (Tokushima, Japan). A 3D-printed scaffold was designed using medical image-computing software based on micro-computer tomography (CT) (R_mCT2 FX, Rigaku Corp., Tokyo, Japan) images of a 24-week-old rabbit femur (Japan SLC Inc., Hamamatsu, Japan). Based on these data, scaffolds with a diameter of 4 mm and a height of 6 mm were molded using a 3D printer (SZ-1100, SK Fine Co. Ltd., Shiga, Japan) to emulate the microstructure of the bone (Fig. 1). The 3D-printed scaffold was manufactured as follows: The slurry was prepared by mixing β-TCP or HAP with UV curing resin (SK Fine Co. Ltd., Shiga, Japan). The slurry was applied to each 70-μm-thick thin layer using a 3D printer and irradiated with a UV laser to create a 3D-printed scaffold. The uncured slurry was then removed from the scaffold. The 3D-printed scaffold was heated to remove the cured resin (500 °C for 6 h) and sintered with β-TCP or HAP (1100 °C for 3 h), and then the temperature was decreased to room temperature by natural cooling in the electric furnace (FUH732PA, ADVANTEC, Tokyo, Japan). Subsequently, the scaffolds were sterilized by dry heating (180 °C, 60 min, MOV-212S, SANYO, Gunma, Japan). Because the cortical bone is a dense body, bone pores were created at intervals of 200 μm to ensure connectivity with the interior.

### Animal experiments

**Fig. 2:**
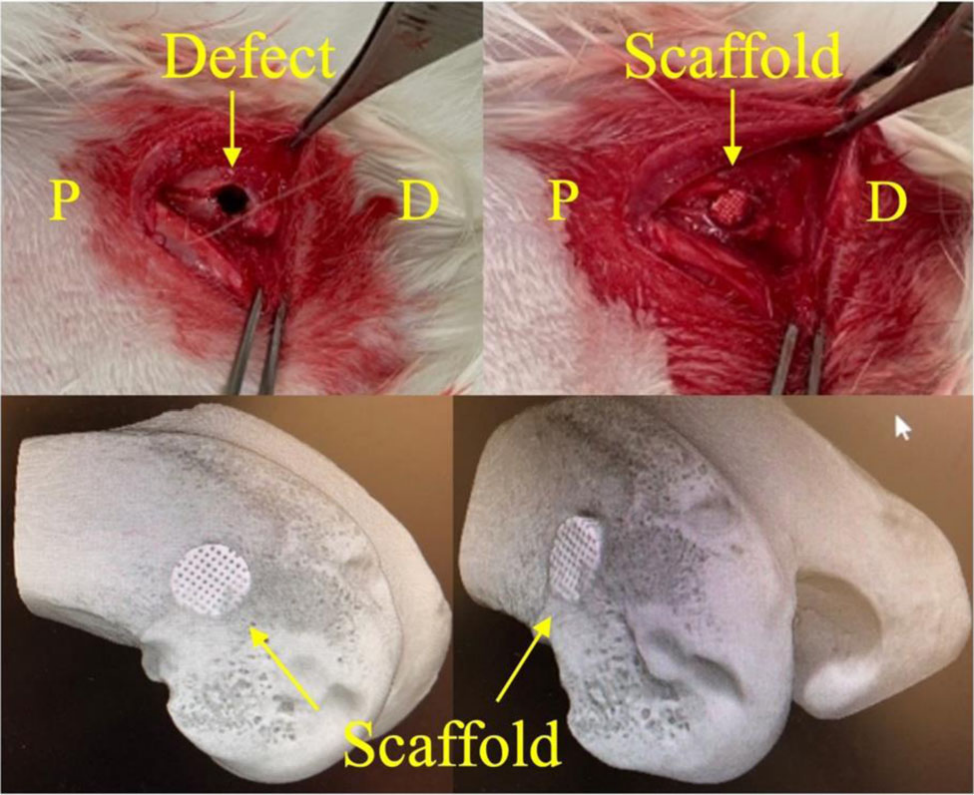
The upper row shows the surgical sites of the rabbit femur defect model. P, proximal; D, distal. The lower row shows 3D CT images of scaffold implantation in the femoral condyle of the rabbit

The Animal Ethics Committee of our hospital approved this study (approval no. P210504). 24-week-old male New Zealand white rabbits were used. The animals were anesthetized using inhalation anesthesia with isoflurane and O_2_ using a mask, as well as intramuscular administration of medetomidine (0.5 mg/kg), midazolam (2 mg/kg), and butorphanol (0.5 mg/kg). A cylindrical bone defect with a diameter of 4.5 mm and a depth of 8 mm was created in the lateral aspect of the distal femur as previously described [17]. A 3D-printed scaffold was implanted in the right femur (experimental side), whereas the left femur was kept free of implantation (control side). The scaffold was easily molded into the bone defect and did not require fixation (Fig. 2). The animals were euthanized with an overdose of sodium pentobarbital at 4, 8, or 12 weeks postoperatively (n = 5 knees in each group).

### Gross observation

The rabbits were sacrificed for gross observation at 4, 8, and 12 weeks after surgery. The femur healing process was also observed. After harvesting, the samples were carefully observed for evidence of severe inflammation, coloration, and ectopic ossification.

### Micro-CT analysis

**Fig. 3:**
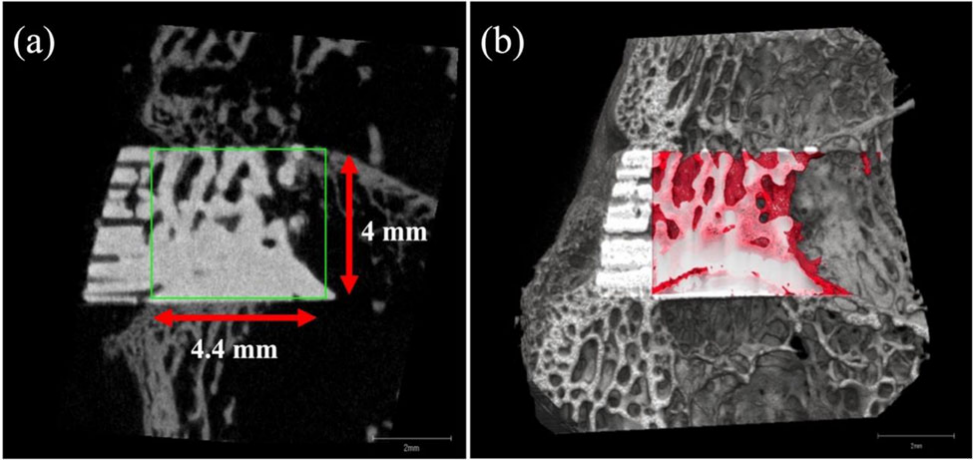
**a** Region of interest (ROI) was defined as 4.4 and 4 mm in a coronal view. Volume/tissue volume, trabecular bone thickness, trabecular number, and trabecular separation were measured within the ROI. **b** The red areas in the 3D CT image indicated new trabecular bone formation

To quantify new trabecular bone formation, we performed micro-CT on the harvested femurs of five animals in each group. After scanning, 3D reconstruction was performed using built-in software. The region of interest was set as the selected area of a cylinder with a diameter of 4 mm and a height of 4.4 mm (Fig. 3a). The bone volume/tissue volume (BV/TV), trabecular bone thickness (Tb.Th), trabecular number (Tb.N), and trabecular separation (Tb.Sp) of the regenerated trabecular bone were calculated from the region of interest using bone microstructure software (TRI/3D-BON-FCS64, Ratoc System Engineering, Tokyo, Japan) (Fig. 3b). The callus bone mineral content was calibrated by scanning hydroxyapatite phantoms of known densities provided by the system manufacturer.

### Histological evaluation

Bone formation was assessed using non-decalcified frozen histology. Kawamoto’s method [18] was used to prepare frozen sections of non-decalcified femurs. At 4, 8, and 12 weeks, the specimens were immersed in a cooled embedding medium (Super Cryoembedding Medium L-1; Section-Lab, Hiroshima, Japan) and quickly frozen in liquid nitrogen to form frozen blocks. Coronal Section (5 μm thick) were mounted on adhesive film (Cryofilm, Section-Lab Co. Ltd., Hiroshima, Japan) with a cryostat (Leica, Nussloch, Germany) and stained with hematoxylin and eosin (HE), toluidine blue, or alkaline phosphatase (ALP) /tartrate-resistant acid phosphatase (TRAP).

### Statistical analysis

The statistical analysis was performed using the SPSS software (SPSS Inc., Chicago, IL, USA). All quantitative data are expressed as mean ± standard deviation. To compare differences between groups, a one-way ANOVA was conducted, and Fisher’s LSD method was used for comparisons between groups. Differences were considered statistically significant if P < 0.05. All statistical tests were two-tailed.

## Results

### Gross observation

All animals tolerated the procedure well, and the wound and soft tissues healed without obvious redness, infection, severe inflammation, coloration, or ectopic ossification. On the control side, the original bone defects were still visible 4 weeks postoperatively but gradually healed over time. At 12 weeks postoperatively, the defect was almost completely covered with cortical bone. On the experimental side, both the β-TCP and HAP groups had a similar gross appearance at 4 weeks postoperatively. The original bone defects were also visible, and the cortical bone portion of the 3D scaffold was clearly exposed. In the β-TCP group, the original bone defect was mostly filled with new bone at 8 weeks postoperatively, the cortical bone area of the 3D scaffold was reduced, and the color of the scaffold became similar to and indistinguishable from the surrounding tissue. The scaffold was clearly visible in the HAP group, although the original bone defect was covered by a thin synovial membrane 8 weeks postoperatively. At 12 weeks postoperatively, the original bone defect was partially covered with regenerated tissue from the margins, but an unfilled area remained in the center of the defect.

### Micro-CT analysis

In both β-TCP and HAP, newly formed bone tissue on the experimental side was detected using micro-CT, whereas almost no bone formation was observed on the control side.

**Fig. 4:**
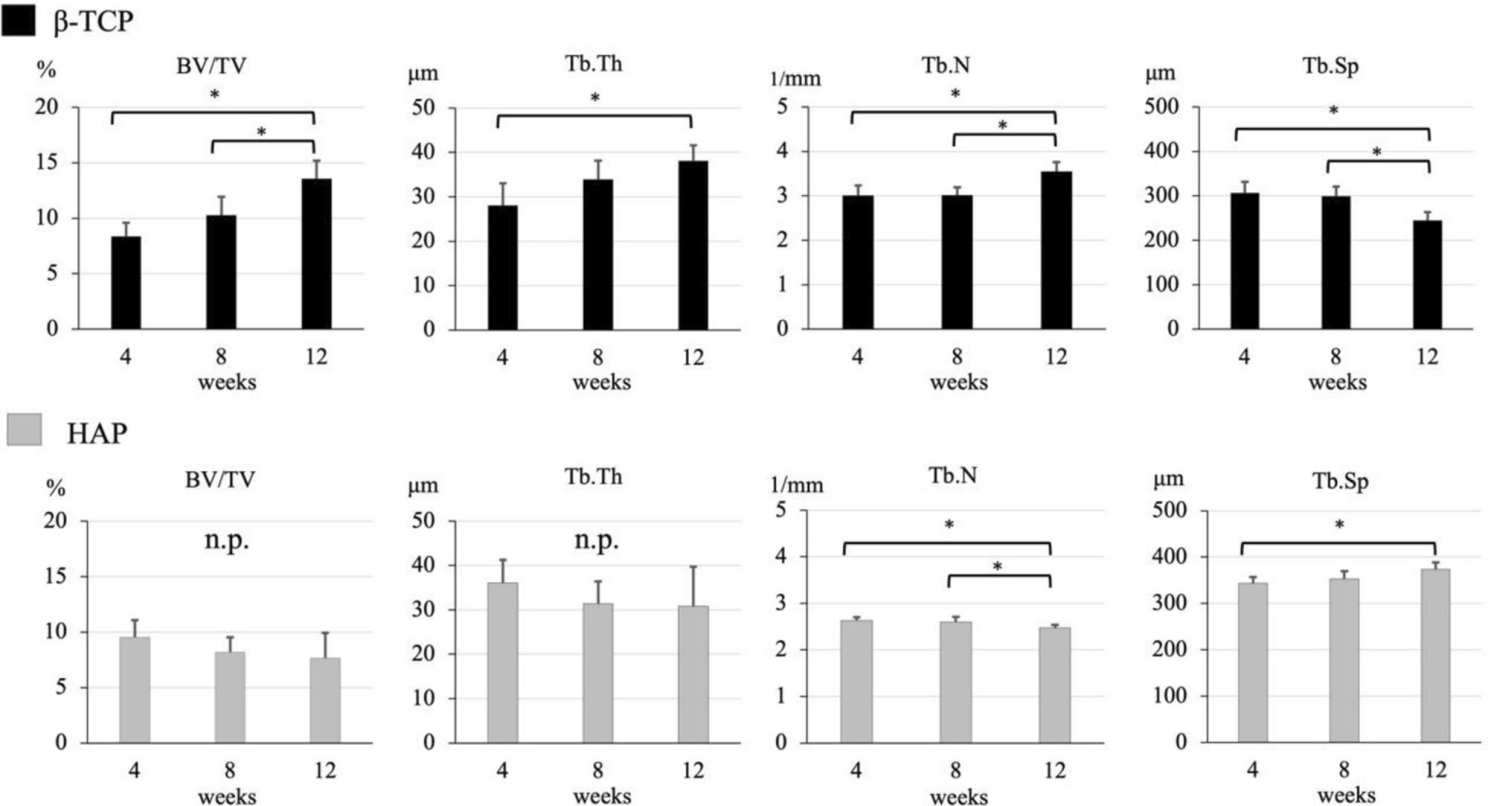
The bone volume/tissue volume (BV/TV), trabecular bone thickness (Tb.Th), trabecular number (Tb.N), and trabecular separation (Tb.Sp) at 4, 8, and 12 weeks. The upper row is β-TCP group, and the lower row is the HAP group

In β-TCP group, the BV/TV, Tb. Th, and Tb. N measured at 4, 8, and 12 weeks postoperatively gradually increased (BV/TV: at 4 weeks 8.38 ± 1.21% versus at 8 weeks 10.25 ± 1.67%; p = 0.053, at 4 weeks versus at 12 weeks 13.56 ± 1.65%; p = 0, at 8 weeks versus at 12 weeks; p = 0.011, Tb. Th: at 4 weeks 28.08 ± 4.97 μm versus at 8 weeks 33.93 ± 4.22 μm; p = 0.055, at 4 weeks versus at 12 weeks 38.14 ± 3.49 μm; p = 0.005, at 8 weeks versus at 12 weeks; p = 0.081, Tb. N: at 4 weeks 3.01 ± 0.22 1/mm versus at 8 weeks 3.02 ± 0.18 1/mm; p = 0.484, at 4 weeks versus at 12 weeks 3.55 ± 0.21 1/mm; p = 0.004, at 8 weeks versus at 12 weeks; p = 0.002), while the Tb. Sp reduced over time (at 4 weeks 306.63 ± 24.45 μm versus at 8 weeks 299.25 ± 21.38 μm; p = 0.331, at 4 weeks versus at 12 weeks 244.71 ± 18.54 μm; p = 0.002, at 8 weeks versus at 12 weeks; p = 0.002) (The upper part of Fig. 4).

In the HAP group, no differences were observed among time points of the BV/TV (at 4 weeks, 9.52 ± 1.57% versus at 8 weeks 8.16 ± 1.37%; p = 0.114, at 4 weeks versus at 12 weeks 7.64 ± 2.29%; p = 0.105, at 8 weeks versus at 12 weeks; p = 0.351) and Tb.Th (at 4 weeks 36.08 ± 5.22 μm versus at 8 weeks 31.37 ± 5.06 μm; p = 0.115, at 4 weeks versus at 12 weeks 30.80 ± 8.91 μm; p = 0.168, at 8 weeks versus at 12 weeks; p = 0.457). Tb. N gradually reduced (at 4 weeks 2.63 ± 0.07 1/mm versus at 8 weeks 2.60 ± 0.11 1/mm; p = 0.318, at 4 weeks versus at 12 weeks 2.47 ± 0.06 1/mm; p = 0.004, at 8 weeks versus at 12 weeks; p = 0.035), while the Tb. Sp increased over time (at 4 weeks 343.85 ± 13.12 μm versus at 8 weeks 353.50 ± 16.41 μm; p = 0.19, at 4 weeks versus at 12 weeks 373.65 ± 15.22 μm; p = 0.009, at 8 weeks versus at 12 weeks; p = 0.055) (the lower part of Fig. 4).

### Histological evaluation

**Fig. 5:**
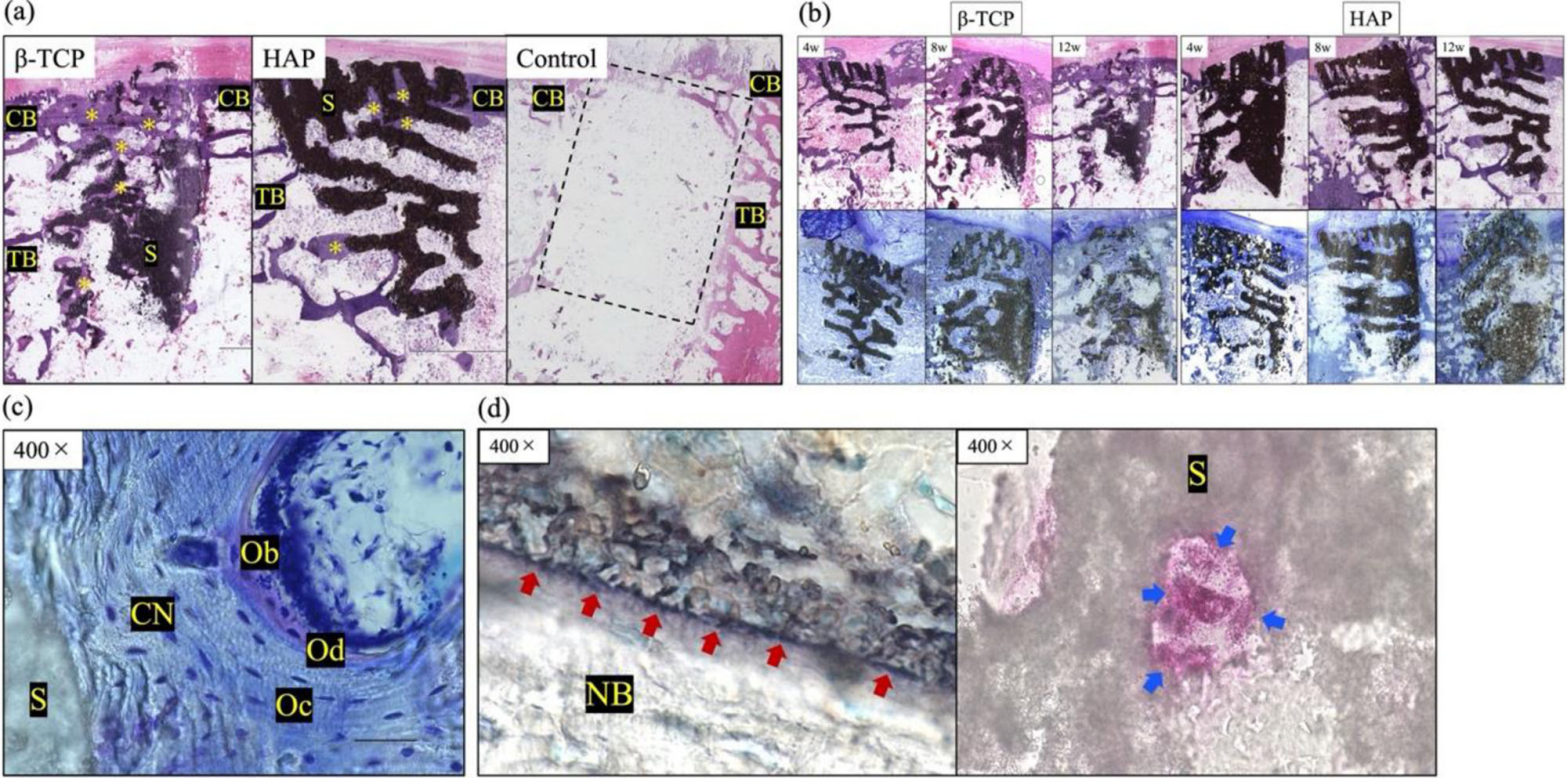
**a** Histological sections of β-TCP, HAP, and control with hematoxylin and eosin (HE) staining at 12 weeks. The black structure represents the scaffold, and the pale purple area along the structure represents the new bone. No new bone was observed in the blank control group (dotted rectangle). *, new bone; CB cortical bone, TB trabecular bone; S, scaffold. **b** Histological sections with HE and toluidine blue staining at 4, 8, and 12 weeks after implantation on the experimental side. The black structure is a scaffold of β-TCP or HAP. The β-TCP scaffold is resorbed over time and replaced by new bone, whereas the HAP remains in almost its original form at 12 weeks. In both groups, new bone formation was observed in the interior of the scaffolds after 4 weeks. **c** Histological sections of β-TCP with toluidine blue staining at 8 weeks in the high-magnification field. CN, new calcified bone; Oc, Osteocyte; Ob, osteoblast; Od, osteoid; S, scaffold. **d** Histological sections of β-TCP with ALP (left) and TRAP (right) staining at 4 weeks. Red arrows indicate osteoblasts (left), and blue arrows indicate osteoclasts (right). S, scaffold; NB, new bone

In the bone-defect group, although cortical bone bridge formation was observed, HE staining at 12 weeks postoperatively showed no new bone in the trabecular bone region (Fig. 5a).

Histological sections of the experimental side stained with HE and toluidine blue are shown in Fig. 5b. The 3D-printed scaffolds maintained their original form for 4 weeks postoperatively. New bone formation was confirmed from the existing bone toward the center of the scaffold. Eight weeks postoperatively, new bone tissue invaded the deep area of the scaffold, and the scaffold was partially degraded. Osteoblasts and a bone matrix with a laminated structure were observed in the high-magnification field (Fig. 5c). In β-TCP, the scaffold was further degraded 12 weeks postoperatively, and cartilage matrix calcification and woven bone formation were observed. In HAP, the 3D-printed scaffold retained most of its original shape, even 12 weeks postoperatively. ALP staining revealed that many active osteoblasts surrounded the newly formed trabecular structures. Osteoclasts were observed by TRAP staining at all the time points (Fig. 5d).

## Discussion

In the current study, we observed that the cells involved in bone metabolism adhered, spread, and proliferated on our newly designed 3D-printed scaffold with a bone microstructure. Therefore, it is suggested that this scaffold has sufficient bioactivity to induce new bone formation and could be expected to be a more useful artificial bone than the existing version.

In recent years, the performance of artificial bones has significantly improved, and the fields of applications are expanding. Researchers have also been investigating the use of 3D printing technology to create customized scaffolds tailored to the specific needs of individual patients. An ideal material for scaffold development should possess biocompatibility and feature a complex framework that replicates the intricate structure of natural tissues [19]. Scaffolds must provide favorable settings that encourage the formation of functional tissues in various forms, including irregular and complicated shapes, while limiting the risks of toxicity and inflammation [20]. In this study, we focused on reproducing the microstructure of the biological bone. In the trabecular bone area of this scaffold, the presence of new bone in the interior at an early stage suggested a good influx of tissue fluid and cells, and bone ingrowth was expected. Normally, most of the ossification process in the scaffold starts from the periphery and gradually creeps up to the center [21]; however, in the initial stage, new bone is also formed in the center of the scaffold. In addition, osteoclasts were observed throughout the scaffold at 4 weeks, suggesting that cell influx and adhesion occurred. Our scaffolds have a natural trabecular bone structure, which may facilitate biological remodeling during osteogenesis and heal bone defects without severe inflammation, coloration, or ectopic ossification. In addition, reproducing the cortical bone using a 3D printer is expected to increase the strength of the scaffold. Namely, the problem of fragile scaffold strength, especially in large bone defects, is a drawback, and a scaffold that reproduces the cortical bone may solve this problem.

HAP is a naturally occurring mineral found in the bone and is often used as a bone graft substitute because of its biocompatibility and similarity to the mineral components of biological bone [3]. It has high osteoconductivity, meaning that it can support the growth of new bone tissue by providing a scaffold for bone cells to attach to and proliferate [22, 23]. It also has a low resorption rate, which means that it can remain in the body for an extended period, providing long-term support for bone regeneration but sometimes leading to a risk of infection [24]. β-TCP has a similar chemical composition to HAP but is more resorbable, meaning it can be broken down by the body and replaced with new bone tissue, but it also has the disadvantage of being difficult to use in load-bearing areas. Although this study suggests that β-TCP ideally promotes bone formation better than HAP, both HAP and β-TCP are valuable materials for bone regeneration and can be used in a variety of situations, depending on the specific needs of the patient and the nature of the bone defect. Further investigation is needed to explore the potential of enhancing the 3D β-TCP scaffold by replicating both cortical and cancellous bone, thereby increasing its strength and regulating its resorbability. This holds promise for the development of improved scaffolds.

This study has some limitations. First, because the purpose of this study was to investigate the bioactivity of the 3D scaffold, the control group had bone defects; however, it might be better to evaluate the performance of this scaffold in comparison with commercially available artificial bone. Second, mechanical tests could not be performed because of the partial bone defect model. We intend to use a segmental bone defect model as a developmental experiment in the future. Third, as we focused on the macrostructure in this study, we did not conduct in vitro studies to evaluate the cell adhesion and osteogenic differentiation potential of the calcium phosphate materials.

## Conclusions

In the current study, we observed the cells involved in bone metabolism adhering, spreading, and proliferating on a newly designed 3D-printed scaffold with a bone microstructure. The scaffold demonstrated sufficient bioactivity and the ability to induce new bone formation when implanted, leading to the expectation that it may be a more effective artificial bone than the existing versions. This study demonstrated new bone formation in a 3D-printed calcium phosphate scaffold with bone microstructure.
